# Averaging, not internal noise, limits the development of coherent motion processing

**DOI:** 10.1016/j.dcn.2014.07.004

**Published:** 2014-08-01

**Authors:** Catherine Manning, Steven C. Dakin, Marc S. Tibber, Elizabeth Pellicano

**Affiliations:** a Centre for Research in Autism and Education (CRAE), Institute of Education, University of London, 55-59 Gordon Square, Institute of Education, London WC1H 0NU, UK; b UCL Institute of Ophthalmology, University College London, Bath Street, London EC 1V9, UK; c NIHR Biomedical Research Centre at Moorfields Eye Hospital, 162 City Road, London EC 1V 2PD, UK

**Keywords:** Visual development, Motion processing, Direction discrimination

## Abstract

The development of motion processing is a critical part of visual development, allowing children to interact with moving objects and navigate within a dynamic environment. However, global motion processing, which requires pooling motion information across space, develops late, reaching adult-like levels only by mid-to-late childhood. The reasons underlying this protracted development are not yet fully understood. In this study, we sought to determine whether the development of motion coherence sensitivity is limited by internal noise (i.e., imprecision in estimating the directions of individual elements) and/or global pooling across local estimates. To this end, we presented equivalent noise direction discrimination tasks and motion coherence tasks at both slow (1.5°/s) and fast (6°/s) speeds to children aged 5, 7, 9 and 11 years, and adults. We show that, as children get older, their levels of internal noise reduce, and they are able to average across more local motion estimates. Regression analyses indicated, however, that age-related improvements in coherent motion perception are driven solely by improvements in averaging and not by reductions in internal noise. Our results suggest that the development of coherent motion sensitivity is primarily limited by developmental changes within brain regions involved in integrating motion signals (e.g., MT/V5).

## Highlights

• Motion processing abilities develop gradually through childhood.
• This lengthy development could be due to local noise and/or poor averaging.
• 5–11-year-olds and adults performed equivalent noise and motion coherence tasks.
• Through childhood, internal noise reduces and averaging increases.
• Yet, only improved averaging explains developments in motion coherence sensitivity.

## 1 Introduction

The processing of motion is a critical part of visual development, allowing children to track moving objects with their eyes, to reach for and grasp objects that are in motion, and to navigate within a dynamic world. Motion processing contributes to a range of elementary visual functions including the segmentation of scenes into different objects and surfaces, the perception of depth, the registration of trajectories and the identification of objects. Often, it is important to combine motion information across space, for example in order to determine the overall direction of a flock of birds, each of which will be following a different motion trajectory. This ability – termed *global motion processing* – is typically tested experimentally using the motion coherence paradigm (Newsome and Paré, 1988), which requires observers to judge the direction of coherently moving dots in the presence of randomly moving noise dots.

Given the importance of motion processing in visual development, it is perhaps unsurprising that some aspects of motion processing (e.g., directional selectivity) develop early in life (Wattam-Bell, 1991, Wattam-Bell, 1992; see Braddick et al., 2003, for review). However, other types of visual motion processing follow a protracted development and only reach adult-like levels by mid-to-late childhood. For example, the minimum speed required to support perception of motion-defined form and the maximum displacement supporting perception of movement mature by around 7–8 years (Hayward et al., 2011, Parrish et al., 2005), motion coherence thresholds reach adult-like levels between 10 and 14 years (Gunn et al., 2002, Hadad et al., 2011) and speed discrimination abilities are not yet fully adult-like by 11 years (Manning et al., 2012). Such motion processing abilities rely primarily on the dorsal pathway (Milner and Goodale, 1995), which originates from motion-sensitive neurons in area V1, and projects to extrastriate areas including MT/V5. While neurons in V1 can signal the presence of local motion (Hubel and Wiesel, 1962), neurons in V5 play a key role in global motion processing, as they have larger receptive fields capable of integrating inputs from V1 (Mikami et al., 1986).

Adult studies of visual motion processing suggest the existence of at least two distinct systems tuned to different ranges of speed (Burr et al., 1998, Edwards et al., 1998, Thompson et al., 2006; also see review by Burr and Thompson, 2011), which may follow different developmental trajectories in the maturing brain. Hayward et al. (2011) reported greater immaturity in sensitivity to coherent motion at the slowest speed tested (0.1°/s) compared to faster speeds of 0.9 and 5°/s. Also, in a speed discrimination task, Manning et al. (2012) reported a more gradual development of thresholds for slow (1.5°/s) than fast (6°/s) speeds. However, Hadad et al. (2011) did not find different rates of development for motion coherence thresholds measured with random dot stimuli moving at 4°/s and 18°/s. Together, this research suggests that motion processing for intermediate and fast speeds may follow similar rates of development, but that processing of much slower speeds (e.g., 0.1 and 1.5°/s) may develop more slowly.

Global motion processing abilities in childhood are generally thought to be limited by poor integration of local motion cues over space (e.g., Ahmed et al., 2005, Hadad et al., 2011, Manning et al., 2012). Such integration is believed to occur in higher-order areas of the motion processing hierarchy, such as in area MT/V5 (Born and Tootell, 1992, Britten et al., 1992). Yet performance on tasks traditionally used to assess global motion processing (i.e., motion coherence paradigms; Newsome and Paré, 1988) is not limited *solely* by global integration. Such tasks are likely limited not only by an observer's ability to globally pool the motion of individual dots across space, but also by their ability to estimate the local motion direction of each dot (Barlow and Tripathy, 1997), and by their ability to segment the signal dots from the masking noise (Dakin et al., 2005, Tibber et al., 2014; Webster et al., 2011).

Increased neural variability would lead to imprecision in estimating individual dot directions, which, when pooled, could lead to elevated motion coherence thresholds. This neural variability has been termed ‘internal noise’, and has many potential sources, including photon noise, variability in the firing of action potentials, and variability in synaptic transmission (Faisal et al., 2008). Through development, neurons in area V1 undergo extensive synaptic pruning (Garey and de Courten, 1983; Huttenlocher et al., 1982; Huttenlocher and de Courten, 1987), and the bandwidths of direction-selective cells reduce with age (at least in the primate brain, Hatta et al., 1998). It is possible that such developmental changes might be manifest as reduced internal noise with age.

**Fig. 1:**
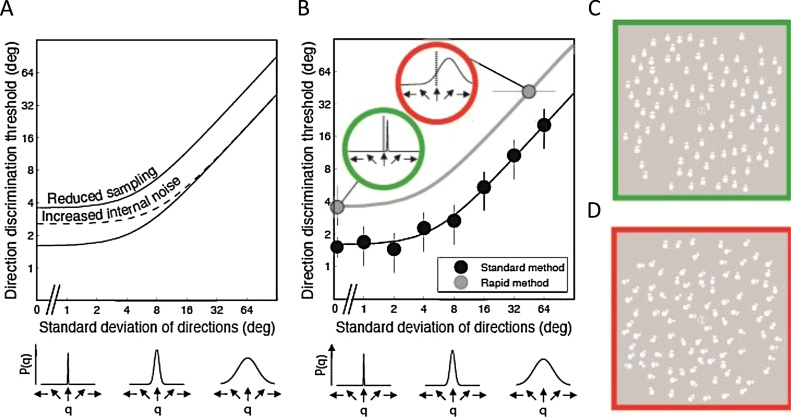
(A) Equivalent noise functions relating direction discrimination thresholds to the standard deviation of dot directions (i.e., external noise). Lower sampling is represented by an equivalent noise function that is shifted vertically upwards, whilst higher levels of internal noise require more external noise to be added before thresholds increase. (B) The black circles and curve represent the standard equivalent noise paradigm where direction discrimination thresholds are measured at multiple levels of external noise. Large standard deviations of dot directions reflect high external noise in the stimulus. The grey circles and curve are derived using a rapid version of the equivalent noise paradigm, which measures performance at two maximally informative noise levels. In the ‘*no noise’* condition, there is no external noise (i.e., the standard deviation of dot directions is 0°) and the threshold is taken as the finest direction discrimination possible. In the ‘*high noise’* condition, we measure the maximum noise that can be tolerated when the observer is judging if the pattern is moving either +45° or −45° of vertical. (C) Example of a stimulus in the ‘*low noise’* condition, where the mean direction of dots is +4°, and the standard deviation of directions is 0°. (D) Example of a stimulus in the ‘*high noise’* condition, where the mean direction of dots is +45°, and the standard deviation of dot directions is 45°. Arrows are provided for illustrative purposes only, to represent the direction of motion.

The traditional motion coherence paradigm cannot distinguish between local and global limits to motion perception and has hence obscured our understanding of what limits global motion processing during development (and in a variety of neurodevelopmental disorders; Dakin and Frith, 2005). To address this issue, the current study used the equivalent noise paradigm (Barlow, 1956, Pelli, 1990) to determine whether local or global processing limits motion coherence sensitivity in development. The equivalent noise paradigm is based on comparing human performance to that of an ideal observer that is limited both by additive internal noise and by how completely it samples the information available from the stimulus (Pelli, 1990). When equivalent noise analysis is applied to direction discrimination (Dakin et al., 2005), *internal noise* maps onto the precision with which individual motion directions are estimated and *sampling* represents an estimate of the effective number of local motion directions that are globally pooled (or averaged). Whereas motion coherence stimuli contain both signal dots and randomly moving noise dots, equivalent noise stimuli contain dots whose directions (on any one trial) are sampled from a single Gaussian distribution (Dakin et al., 2005). The standard deviation of this distribution is varied across conditions, in order to manipulate the level of stimulus variability (or ‘external noise’; see Fig. 1A).

In the equivalent noise task, the observer is asked to discriminate the mean direction of dots, and the performance measure is the smallest difference in direction from a fixed reference direction (e.g., upwards) that observers can reliably report. With no directional variance (i.e., when the standard deviation is 0° and all elements move in the same direction), the observer's performance is limited both by internal noise and sampling. Consequently, small amounts of extra external noise have little effect on thresholds, as it is swamped by the observer's own internal noise. However, as the level of external noise is increased, a point is reached where the external noise exceeds the internal noise inherent in the system, and thresholds start to increase with the addition of further external noise. An equivalent noise function can be fit to these data to derive estimates of the individual's internal noise and sampling (see Fig. 1A).

As thresholds are measured across a range of external noise levels, the equivalent noise method typically requires several thousand trials, making it unsuitable for investigating the visual abilities of children, who may get bored and become inattentive. However, a more efficient equivalent noise procedure has been developed, which provides reliable estimates of internal noise and sampling in fewer than 100 trials (Tibber et al., 2014). In this novel method, two highly informative points on the equivalent noise function are probed (see grey line, Fig. 1B). In one condition (‘*no noise’*, Fig. 1C), the standard deviation of dot directions is 0°, and an adaptive staircase procedure is used to estimate the finest direction discrimination possible. In the other condition (‘*high noise’*, Fig. 1D), an adaptive staircase procedure estimates how much directional variability can be tolerated while discriminating a large (±45°) directional offset. As these thresholds have confidence intervals that lie in orthogonal planes, the fit of the equivalent noise function is efficiently constrained to provide reliable estimates of internal noise and sampling.

Here, we used Tibber et al.’s rapid version of the equivalent noise direction integration paradigm alongside a traditional motion coherence task to investigate the factors limiting the development of global motion processing. These methods allowed us to investigate (1) how internal noise and sampling develop, and (2) the extent to which changes in these factors impact upon a commonly used measure of global motion processing, namely motion coherence thresholds. Due to the possibility of distinct developmental trajectories for different speeds (Hayward et al., 2011, Manning et al., 2012), equivalent noise and motion coherence tasks were presented at two stimulus speeds: slow (1.5°/s) and fast (6°/s).

It is commonly assumed that motion coherence thresholds are limited by poor integration of local motion information (e.g., Hadad et al., 2011). We therefore hypothesised that sampling would increase with age and that this would contribute to age-related reductions in motion coherence thresholds. Deriving hypotheses about the development of internal noise was less straightforward. Indeed, some researchers have noted that children have high trial-to-trial behavioural variability which decreases with age (e.g., Williams et al., 2005), where higher behavioural variability is thought to reflect higher neuronal variability (i.e., noise; Fox et al., 2007, but see also Beck et al., 2012). Additionally, Skoczenski and Norcia (1998) measured internal noise in infants using an equivalent noise technique with visually evoked potential (VEP) responses and reported that high levels of internal noise in infancy limited contrast sensitivity. Similarly, Buss et al. (2006) demonstrated increased levels of internal noise in children aged 5–10 years compared to adults, with children being less susceptible to the effects of external noise (roving intensities) in an auditory intensity discrimination task. Alternatively, some have suggested that neuronal variability in fact *increases* with age from 8 years to adulthood, as measured by trial-by-trial EEG variability (McIntosh et al., 2008). Evidently, internal noise has been measured in a range of different ways and it is not yet clear how these measures relate to each other. In the current study, we therefore aimed to investigate *how* internal noise and sampling change through childhood for a direction integration task and to determine whether such changes limit the development of motion coherence perception.

## 2 Materials and methods

### 2.1 Participants

Five groups of participants were tested, with 21 5-year-olds (*M* = 5 years; 4 months, range 4; 10–5; 10, 14 females), 27 7-year-olds (*M* = 7 years; 3 months, range 6; 7–7; 10, 11 females), 25 9-year-olds (*M* = 9 years; 2 months, range 8; 8–9; 9, 11 females), 20 11-year-olds (*M* = 11 years; 3 months, range 10; 8–11; 9, 14 females) and 30 adults (*M* = 26 years; 9 months, range 21; 5–35; 10, 15 females) included in the final dataset. Children were recruited from schools in the South East of England. Normal or corrected-to-normal visual acuity was confirmed by binocular testing with letter acuity tests using optical corrections where necessary. Normal acuity was defined as a binocular acuity of 6/9 or better for 5- and 7-year-olds (because acuity is still maturing in this age range; Adams and Courage, 2002, Ellemberg et al., 1999) and 6/6 or better for 9- and 11-year-olds and adults.

An additional nine 5-year-olds were excluded from the dataset, with one child failing to pass the visual acuity screening, one failing to reach criterion (see Section 2.3.1), three not performing significantly above chance in the catch trials (see Section 2.6.2) and four obtaining motion coherence thresholds above 100%, indicating an inability to perform the task. One additional 7-year-old could not complete the motion coherence task. An additional two 9-year-olds, one 11-year-old and one adult were excluded from the dataset due to diagnoses of developmental conditions.

### 2.2 Apparatus and stimuli

The stimuli were presented using MATLAB (The Mathworks Ltd.) using elements of the Psychophysics Toolbox software (Brainard, 1997, Kleiner et al., 2007, Pelli, 1997). Stimuli were displayed on a Dell Precision M4600 laptop at a frame rate of 60 Hz and a pixel resolution of 1366 × 768.

**Fig. 2:**
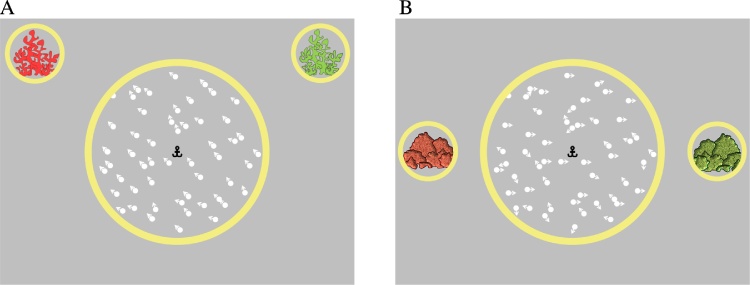
Schematic representation of stimuli presented in the ‘high noise’ condition of the equivalent noise task (A) and the motion coherence task (B). The anchor-shaped fixation point, central yellow aperture and green (left) and red (right) “reefs” or “rocks” remained on the screen throughout the trial.

A yellow-bordered circular aperture (diameter = 15°) and anchor-shaped fixation point (0.57 × 0.57°) were presented against a grey background with a luminance of 30 cd/m^2^ (see Fig. 2). Two smaller yellow-bordered circular apertures (diameter = 6.12°) were presented to the left and right of this, serving as reference points for the reporting of motion direction. In the equivalent noise task, the left and right apertures were presented in the top corners of the screen and contained images of red and green reefs, respectively (see Fig. 2A). In the motion coherence task, the left and right apertures were presented halfway down the screen, containing images of red and green rocks, respectively (see Fig. 2B).

The stimuli were comprised of 100 randomly positioned white dots (58.7 cd/m^2^), each with a diameter of 0.44°, drifting for 400 ms within the central aperture. Dot positions were updated every 3 frames with displacements of 0.075° and 0.3° in the slow (1.5°/s) and fast (6°/s) conditions, respectively. Dots were allowed to overlap and were not limited in their lifetime.

### 2.3 Procedure

Participants completed an equivalent noise task and a motion coherence task in each of two speed conditions: slow (1.5°/s) and fast (6°/s). In the equivalent noise task, dot directions were randomly sampled from a wrapped normal distribution with a specified mean and standard deviation. The equivalent noise task consisted of two interleaved conditions that probed two informative points on the equivalent noise function to constrain the fit of the model (see Fig. 1B). In the ‘*no noise’* condition, the standard deviation of dot directions was fixed at 0° (i.e., all dots moved in the same direction), while the mean direction of the dots was varied (leftward or rightward of vertical) to find the finest direction that could be discriminated 84% of the time in the absence of stimulus noise (corresponding to the mean plus one standard deviation in a cumulative normal distribution). In the ‘*high noise’* condition, the mean direction of dots was fixed at 45° leftwards or rightwards of vertical-upwards motion, and the standard deviation of dot directions was varied to find the maximum level of noise that could be tolerated whilst identifying the signal direction with 84% accuracy.

The equivalent noise task was presented as “The Hungry Fish Game”. Participants judged whether a shoal of “fish” was “swimming” towards the red (left) or green (right) reef to find their food. Children were told that sometimes the fish all moved in the same direction (‘*no noise*’) and sometimes the fish moved in different directions (‘*high noise*’), in which case they had to determine the overall (i.e., average) direction. To aid motivation, children were told that they were competing against a cartoon character, “Scuba Sam”.

In the motion coherence task, a proportion of dots moved coherently in a single direction (90° leftward or rightward of vertical) while the remaining dots moved in random directions. The task was presented within the context of “The Shark Attack Game”. Participants were asked to judge whether the shoal of “fish” was “swimming” towards the red (left) or green (right) rocks to hide from the “shark”. Children were told that the “fish” sometimes “panicked” when they saw the “shark”, causing them to go in different directions. To enhance motivation, children were told that they were competing against the “shark”.

Each equivalent noise and motion coherence task consisted of three levels: a combined demonstration and criterion phase (‘level 1’), a practice phase (‘level 2’), and a threshold estimation phase (‘level 3’). In all levels in both tasks, direction (leftward or rightward of vertical) was randomised on each trial.

#### 2.3.1 Demonstration and criterion phase

The experimenter explained each task to participants within the context of four demonstration trials, two of which were designed to be ‘easy’, and two of which were ‘slightly harder’. In the equivalent noise task, two of the trials demonstrated the ‘*no noise’* condition, and two demonstrated the ‘*high noise’* condition. Next, participants were presented with up to 20 criterion trials. In the equivalent noise task, ‘*no noise*’ stimuli were presented with a direction of 45° leftward or rightward of vertical. In the motion coherence task, dots moved with 100% coherence 90° leftward or rightward of vertical. Participants who failed to reach a criterion of four consecutive correct responses within 20 trials were given a short version of the task and excluded from analysis (*n* = 1). Children responded either verbally or by pointing, with the experimenter relaying the response to the computer. Visual and verbal feedback and encouragement were provided.

#### 2.3.2 Practice phase

Eight practice trials were presented in a fixed order for each task with increasing difficulty. In the equivalent noise task, four ‘*no noise*’ stimuli and four ‘*high noise*’ stimuli were presented in alternating order. Participants received feedback as before, but there was no criterion for proceeding to the next phase.

#### 2.3.3 Threshold estimation phase

Both the equivalent noise and motion coherence tasks employed the QUEST adaptive staircase method (Watson and Pelli, 1983). In the equivalent noise task, two staircases (75 trials each) were interleaved for each of the ‘*no noise*’ and ‘*high noise*’ conditions. In the ‘*no noise*’ condition, the QUEST function tracked the basic direction offset threshold in the absence of noise. In the ‘*high noise’* condition, the mean direction of motion was set to ±45° and QUEST tracked the maximum level of noise that could be tolerated whilst discriminating the mean direction. An additional 15 catch trials were interleaved randomly, presenting stimuli identical to those used in the criterion phase. This yielded 165 trials in total for each speed condition.

In the motion coherence task, a single QUEST staircase of 75 trials tracked the minimum coherence level required for accurate (84% correct) direction discrimination. As in the equivalent noise task, there were an additional 15 catch trials, which presented stimuli used in the criterion phase. This resulted in 90 trials in total for each speed condition. Trials were divided into four blocks of equal length for each condition of each task. When the end of a block was reached, participants were shown a simulated graph of the “points” they and their “opponent” (“Scuba Sam” or the “shark”) had attained. These points were randomly jittered around a fixed set of values to minimise reward and motivation effects on threshold estimates.

### 2.4 Eyetracking

To establish whether developmental differences in task performance could be accounted for by differences in ability to maintain fixation, we used a Tobii X2-30 Compact eyetracker mounted onto the screen to collect fixation data for a subset of participants, including 12 five-year-olds, 17 seven-year-olds, 11 nine-year-olds, 9 11-year-olds and 10 adults. A five-point calibration procedure was conducted before the introductory phase and fixation data were sampled at a rate of 40 Hz during stimulus presentation in the threshold estimation phase.

### 2.5 General procedure

The procedure was approved by the Institute of Education's Faculty Research Ethics Committee. All adult participants and parents of child participants gave their informed consent. Children provided verbal assent. Participants were seen individually for two sessions lasting approximately 25 min, each consisting of one equivalent noise and one motion coherence task. The order of presentation of conditions was counterbalanced across participants. Participants were seated in a dimly lit room 51 cm from the monitor which they viewed binocularly using a chin-rest. They were instructed to maintain central fixation throughout stimulus presentation, which the experimenter monitored, providing reminders to maintain fixation and only initiating trials when the participant was attending. Participants were each given a ‘Submarine Log Book’ with which they recorded their progress through the experimental session.

### 2.6 Data analysis

#### 2.6.1 Equivalent noise analysis

The equivalent noise model describes changes in direction discrimination threshold as a function of external noise:

(1) $$\sigma_{obs}^{2}=\frac{\sigma_{\mathrm{int}}^{2}+\sigma_{ext}^{2}}{n_{samp}}$$

where $\sigma_{obs}^{2}$ is the observer's threshold, $\sigma_{\mathrm{int}}^{2}$ is additive internal noise, $\sigma_{ext}^{2}$ is the external noise added to the stimulus, and *n*_samp_ is the effective number of samples used to calculate the mean direction of the stimulus. This approach exploits additivity of variance, whereby internal noise and external noise contribute independently to an observer's direction discrimination threshold.

The equivalent noise task yielded two thresholds: (a) the finest direction discrimination possible with no stimulus noise (‘*no noise*’ condition), and (b) the maximum level of noise that could be tolerated whilst discriminating a large signal offset of 45° (‘*high noise*’ condition). By running Monte Carlo simulations of a model observer's performance across a range of internal noise and sampling levels, Bex et al. have shown that – assuming that a participant's internal noise is negligible at high noise levels – sampling (*n*_samp_) can be estimated from a linear transformation of their maximum tolerable noise threshold (MTN):

(2) $$n_{\text{samp}}=\exp(0.000121*{\text{MTN}}^{2}+0.0357*\text{MTN}-1.8093)$$

As performance at low levels of external noise is determined both by internal noise and sampling, it is possible to use the estimate of *n*_samp_ to compute the level of internal noise, by rearranging Eq. (1). Thus, when external noise is zero ( $\sigma_{ext}^{2}=0$):

(3) $$\sigma_{\mathrm{int}}^{2}=\sigma_{obs}^{2}\times n_{samp}$$

This approach assumes that observers do not change their sampling (or more generally, their strategy) as a function of external noise level. Consistent with this view, the equivalent noise function has been shown to fit direction discrimination data over a wide range of external noise levels (directional variability), under varying stimulus conditions (Dakin et al., 2005). Note that this approach does not assume that observers are necessarily averaging dot directions in the way the model does to make perceptual judgements. No matter how observers perform the task the model will return the *effective* number of samples that are averaged – that is to say that the observer is acting *as if* they were averaging a certain number of dots. Thus all noise and sampling estimates quoted are necessarily effective values since we cannot know the observer's underlying strategy for performing the task.

#### 2.6.2 Data screening and transformation

A lapse rate was calculated as the proportion of incorrect responses to catch trials for each participant for each condition for each task. A binomial test revealed that participants responding incorrectly on 4 or more of the catch trials were not performing significantly above chance. Three five-year-olds were therefore excluded from analyses (see Section 2.1).

Analysis of variance (ANOVA) showed that lapse rates differed significantly across age groups, *F*(4,118) = 9.26, *p* < .01, $\eta_{p}^{2}=.24$ (5-year-olds: *M* = .04, SD = .06; 7-year-olds: *M* = .02; SD = .04, 9-year-olds: *M* = .01, SD = .03; 11-year-olds: *M* = .01, SD = .03; adults, *M* < .01, SD = .01). Post hoc Dunnett *t*-tests comparing each of the age groups with the adult groups revealed that 5-year-olds and 7-year-olds had significantly higher lapse rates than adults (5-year-olds: *p* < .01; 7-year-olds: *p* < .01), whereas the 9- and 11-year-olds did not differ from the adult group (*p* > .05). There was no main effect of task (*p* = .45), although higher lapse rates were found for the slow speed conditions (*M* = .02, SD = .05) than the fast speed conditions (*M* = .01, SD = .03), *F*(1,118) = 15.40, *p* < .01, $\eta_{p}^{2}=.12$. No interactions were significant (*p*s > .05).

To ensure that any age-related and/or speed-related differences in internal noise, sampling or motion coherence thresholds were not a by-product of differences in attention, an ideal observer model was run assuming different levels of lapse rate. Monte Carlo simulations allowed us to model the effect of differing lapse rates on thresholds. We averaged across tasks to get a lapse rate for each observer in each speed condition, and then corrected the thresholds for each observer according to their lapse rate for each speed condition, based on the simulation results.

Next, the internal noise, sampling and motion coherence threshold estimates in each speed condition were assessed for skewness and kurtosis. All measures showed significant positive skew (*p*s < .05) and the majority showed significant kurtosis (*p*s < .05). Consequently, all data were log-transformed. The data were then screened for outliers lying more than three *z* scores from the mean for each age group in each speed condition. No outliers were found in motion coherence thresholds, internal noise or sampling estimates. All of the analyses reported below were conducted with log-transformed, lapse-corrected values.

#### 2.6.3 Fixation analysis

Raw fixation data were (*x*,*y*) coordinates sampled during stimulus presentation in each trial of the threshold estimation phase for left and right eye positions relative to the screen's active display area. The data were initially filtered according to a validity code from 0 (signifying the eye was definitely found) to 4 (signifying the eye was not found). All samples with validity codes of 2 or higher were discarded (Tobii Technology, 2013). The (*x*,*y*) coordinates were then averaged across the left and right eye for analysis. A measure of fixation stability was derived by pooling the standard deviations of fixation locations in *x* and *y* dimensions. The standard deviations were then log-transformed to minimise the effects of skewness and kurtosis.

## 3 Results

### 3.1 Age-related changes in internal noise

**Fig. 3:**
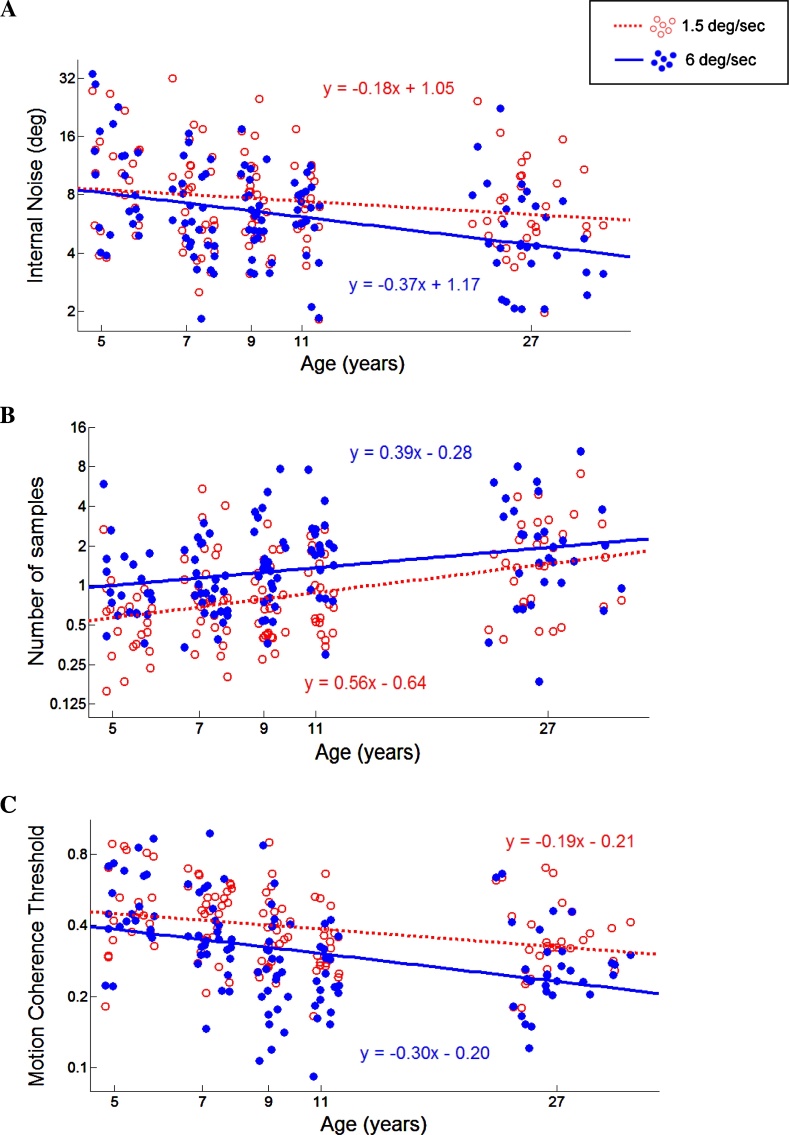
Individual values for internal noise (A), sampling (B) and motion coherence thresholds (C) for slow (1.5°/s) (open red circles) and fast (6°/s) (filled blue circles) conditions as a function of age. Red dashed and blue solid lines represent the line of best fit for the slow and fast conditions, respectively. (For interpretation of the references to color in this figure legend, the reader is referred to the web version of the article.)

Levels of internal noise reduced with age, with 5-year-olds having mean levels of 9.62° and 9.69° in the slow and fast conditions, respectively, which reduced to 6.72° and 4.80° in the adult group. To characterise the rate of developmental changes in estimated internal noise, log internal noise values were plotted as a function of log age and fit with a straight line (Fig. 3A). We then compared the developmental trajectories for slow and fast speeds using the ANCOVA method outlined by Thomas et al. (2009). In this method, within-subjects effects are initially examined using an ANOVA before assessing age-related changes by adding a covariate (as within-subjects effects are masked when a between-subjects covariate is added; Delaney and Maxwell, 1981, Thomas et al., 2009). An initial ANOVA with speed condition (slow, fast) as a within-subjects factor revealed that significantly higher levels of log internal noise were found in the slow (*M* = .87, SD = .24) than the fast condition (*M* = .79, SD = .25), *F*(1,122) = 12.24, *p* < .01, $\eta_{p}^{2}=.09$. Next, an ANCOVA was conducted by adding log age into the model as a covariate. Overall, log internal noise reduced significantly with age, *F*(1,121) = 13.42, *p* < .01, $\eta_{p}^{2}=.10$. Also, there was a significant interaction between log age and speed condition, *F*(1,121) = 4.76, *p* = .03, $\eta_{p}^{2}=.04$, indicating a significantly steeper rate of development in the fast condition than the slow condition.

Dunnett *t*-tests (corrected for multiple comparisons) were conducted to determine when adult-like levels of log internal noise were reached for slow and fast speed conditions. In the slow condition, 5-year-olds had significantly higher log internal noise than adults (*p* = .02) whereas 7-, 9- and 11-year-olds had adult-like levels of log internal noise (*p*s > .05) (5-year-olds: *M* = .98, SD = .25; 7-year-olds: *M* = .86, SD = .27; 9-year-olds: *M* = .87, SD = .23; 11-year-olds: *M* = .83, SD = .22; adults: *M* = .83, SD = .23). Similarly, in the fast condition, 5-year-olds had higher log internal noise than adults (*p* < .01) whereas the older age groups did not (*p*s > .05) (5-year-olds: *M* = .99, SD = .28; 7-year-olds: *M* = .78, SD = .23; 9-year-olds: *M* = .80, SD = .20; 11-year-olds: *M* = .78; SD = .21; adults: *M* = .68, SD = .26).

### 3.2 Age-related changes in sampling

Next, we investigated age-related changes in sampling. As shown in Fig. 3B, sampling increased from 0.51 at age 5 to 1.47 in adults in the slow condition, and from 0.98 to 1.85 in the fast condition. We repeated the ANOVA and ANCOVA analyses using log-transformed levels of sampling as the dependent variable. Higher levels of log sampling were obtained in the fast condition (*M* = −.06; SD = .34) than in the slow condition (*M* = .13; SD = .34), *F*(1,122) = 39.12, *p* < .01, $\eta_{p}^{2}=.24$. When log age was added into the model as a covariate, it was found that log sampling increased across development, *F*(1,121) = 23.32, *p* < .01, $\eta_{p}^{2}=.16$, as predicted. However, there was no interaction between speed condition and log age, *F*(1,121) = 1.88, *p* = .17, suggesting a similar rate of development in slow and fast conditions.

Dunnett *t*-tests revealed that all child groups had lower log sampling compared to adults (*M* = .17, SD = .35) in the slow condition (5-year-olds: *M* = −.29, SD = .28; *p* < .01; 7-year-olds: *M* = −.06, SD = .35; *p* < .01; 9-year-olds: *M* = −.15, SD = .28; *p* < .01; 11-year-olds: *M* = −.06, SD = .28; *p* = .01). In the fast condition, 5-year-olds (*M* = −.01, SD = .28) and 7-year-olds (*M* = −.01; SD = .25) had lower log sampling than adults (*M* = .27; SD = .40) (*p*s<.01) whereas 9-year-olds (*M* = .14, SD = .33) and 11-year-olds (*M* = .23, SD = .30) did not differ significantly from adults (*p*s > .05).

### 3.3 Age-related changes in motion coherence thresholds

Whereas 5-year-olds required, on average, 47% coherent motion in both the slow and fast conditions to reliably report the direction of motion, adults required only 34% and 26% coherent motion in the slow and fast conditions, respectively. The ANOVA and ANCOVA analyses were repeated to characterise developmental changes in log motion coherence thresholds (Fig. 3C). Higher log motion coherence thresholds were found in the slow condition (*M* = −.41, SD = .16) than the fast condition (*M* = −.51, SD = .21), *F*(1,122) = 37.18, *p* < .01, $\eta_{p}^{2}=.23$. Thresholds decreased with log age, *F*(1,121) = 20.50, *p* < .01, $\eta_{p}^{2}=.14$, but there was no significant interaction between speed condition and log age, *F*(1,121) = 2.73, *p* = .10, indicating that sensitivity developed at a similar rate for slow and fast speeds.

Five-year-olds and 7-year-olds had significantly higher log thresholds than adults in both the slow and fast conditions (*p*s < .01), whereas 9- and 11-year-olds showed adult-like levels of performance (*p*s > .05) (5-year-olds: *M*_slow_ = −.33, SD_slow_ = .18, *M*_fast_ = −.32, SD_fast_ = .17; 7-year-olds: *M*_slow_ = −.35, SD_slow_ = .14, *M*_fast_ = −.44, SD_fast_ = .17; 9-year-olds: *M*_slow_ = −.42, SD_slow_ = .15, *M*_fast_ = −.58, SD_fast_ = .22; 11-year-olds: *M*_slow_ = −.48, SD_slow_ = .14, *M*_fast_ = −.63, SD_fast_ = .16; adults: *M*_slow_ = −.47, SD_slow_ = .16, *M*_fast_ = −.58, SD_fast_ = .17).

### 3.4 Relationship between equivalent noise measures and motion coherence thresholds

Our results show that internal noise reduces, and sampling increases, through development, while motion coherence thresholds decrease. Next we sought to investigate whether increasing sensitivity to coherent motion is driven either by internal noise or sampling, or a combination of both. Correlation analyses including all participants revealed no relationship between internal noise and motion coherence thresholds in either slow, *r* = .03, *df* = 122, *p* = .77, or fast, *r* = .08, *df* = 122, *p* = .36, conditions. However, sampling was negatively correlated with motion coherence thresholds in both slow, *r* = −.35, *df* = 122, *p* < .01, and fast, *r* = −.34, *df* = 122, *p* < .01, conditions.

We built a hierarchical regression model on log motion coherence thresholds for each speed condition, with log age added into the model first, followed by sampling and internal noise added in a stepwise manner (see Table 1). In both slow and fast conditions, log age significantly predicted motion coherence thresholds in the first step of the model. When sampling and internal noise were added into the second step of the model, age remained a significant predictor of motion coherence thresholds, and sampling was also a significant predictor in both slow and fast conditions. Internal noise, however, failed to significantly predict coherence thresholds for either speed condition (slow, *β* = .14, *p* = .16, or fast, *β* = .08, *p* = .41), and was therefore excluded from the model in both speed conditions. Step 2 of the model, with both log age and sampling, was a significantly better model than Step 1 of the model in both speed conditions (see Table 1). The resulting model with log age and log sampling significantly predicted log motion coherence thresholds in both slow (*F*(2,120) = 10.63, *p* < .01) and fast (*F*(2,120) = 14.32, *p* < .01) conditions.

**Table 1:** Hierarchical regression analyses on log motion coherence thresholds in the slow (1.5°/s) and fast (6°/s) conditions.

	Slow condition			Fast condition		
	B	SE B	β	B	SE B	β
Step 1						
Constant	−0.21	0.06		−0.20	0.07	
Log age	−0.19	.06	−.29**	−0.30	0.07	−.36**
Step 2						
Constant	−0.30	0.06		−0.25	0.07	
Log age	−0.12	0.06	−.18*	−0.24	0.07	−.29**
Log sampling	−0.13	0.04	−.28**	−0.16	0.05	−.26**

*Note*:

In the slow condition, *R*^2^ = .09, *p* < .01 for Step 1; Δ*R*^2^ = .06, *p* < .01 for Step 2. In the fast condition, *R*^2^ = .13, *p* < .01 for Step 1; Δ*R*^2^ = .06, *p* < .01 for Step 2.

* *p* < .05.

** *p* < 01.

### 3.5 Fixation analysis

**Fig. 4:**
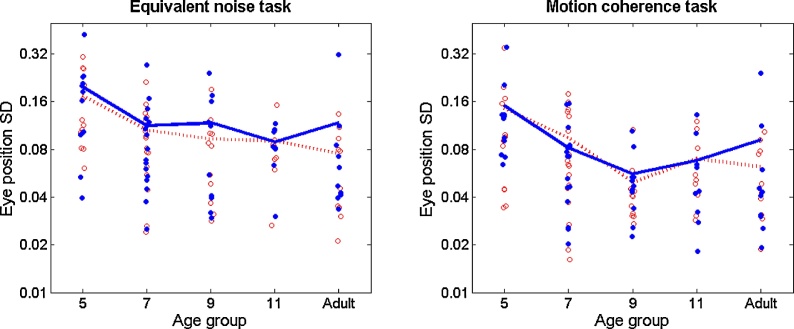
Standard deviations of eye positions in equivalent noise tasks (left panel) and motion coherence tasks (right panel) for slow (1.5°/s) and fast (6°/s) speed conditions. Circles show individual performance (slow: open circles; fast: filled circles) and lines represent mean performance for each age group (slow: red dotted line; fast: blue solid line). Standard deviations were log-transformed for analysis. (For interpretation of the references to color in this figure legend, the reader is referred to the web version of the article.)

Next, we investigated whether there were age-related changes in the ability to maintain fixation and whether these were related to task performance. The standard deviation of participants’ eye positions for each task is shown in Fig. 4. A preliminary ANOVA on standard deviations in the equivalent noise task revealed no main effect of noise condition (‘*no noise*’, ‘*high noise*’) and no interactions with age group or speed condition, and so this factor was not analysed further.

A mixed ANOVA was conducted on the standard deviations with speed (1.5°/s, 6°/s) and task (equivalent noise, motion coherence) as within-participants factors and age group (5-, 7-, 9- and 11-year-olds and adults) as a between-participants factor. There was no main effect of stimulus speed, *F*(1,54) = 1.34, *p* = .25. However, higher standard deviations (i.e., reduced stability) were found in the equivalent noise task (*M* = −1.08, SD = .28) than the motion coherence task (*M* = −1.22, SD = .28), *F*(1,54) = 52.47, *p* < .01, $\eta_{p}^{2}=.49$. There was a significant main effect of age, *F*(4,54) = 4.08, *p* < .01, $\eta_{p}^{2}=.23$. Dunnett *t*-tests revealed that 5-year-olds had significantly larger standard deviations (*M* = −.92, SD = .27) than adults (*M* = −1.27, SD = .28), *p* < .01, whereas the older age groups were not significantly different to adults (7-year-olds: *M* = −1.14, SD = .27; 9-year-olds: *M* = −1.26, SD = .26; 11-year-olds: *M* = −1.19, SD = .23; *p*s > .05). No significant interactions were found between task, speed condition and group (*p*s > .05).

Having found that the youngest children have less stable fixations than older participants, we sought to investigate whether these differences related to internal noise and sampling. Given that higher levels of internal noise and lower sampling are found in the slow condition (Sections 3.1, 3.2), we conducted separate analyses for each speed condition. In the slow condition, fixation standard deviation was related to internal noise estimates, *r* = .28, *df* = 58, *p* = .04, with lower fixation standard deviations (i.e., more stable fixations) being associated with lower internal noise. There was, however, no relationship between fixation standard deviation and sampling, *r* = −.14, *df* = 58, *p* = .29. Similarly, in the fast condition, fixation standard deviation was related to internal noise, *r* = .30, *df* = 58, *p* = .02, but not sampling, *r* = −.05, *df* = 58, *p* = .72. Finally, we investigated the relationship between fixation stability and motion coherence thresholds. Motion coherence thresholds were not related to standard deviation of eye positions in either slow, *r* = .14, *df* = 58, *p* = .31, or fast, *r* = .17, *df* = 58, *p* = .20, conditions.

## 4 Discussion

This study presented an equivalent noise motion integration task alongside a traditional motion coherence task to children aged 5, 7, 9 and 11 years and adults for two speed conditions (slow: 1.5°/s; fast: 6°/s). This method allowed us to characterise both age-related changes in internal noise and sampling and the mechanisms supporting coherent motion processing. While there was considerable individual variability, we found that internal noise estimates reduce through childhood, reflecting improved local processing, and that this is accompanied by an increase in the number of samples the child can use to estimate global motion. Note that the effective number of samples can also be thought of as a measure of multiplicative noise being added to all estimates in the pooling process (i.e., ‘global noise’; Dakin et al., 2005). Although levels of internal noise reduced with age, these did not predict motion coherence thresholds. Instead, developmental increases in motion coherence sensitivity appear to be driven solely by age-related increases in sampling.

Overall, higher levels of internal noise and lower sampling were found in the slow (1.5°/s) condition than the fast (6°/s) condition, which might reflect distinct speed-tuned motion processing systems (e.g., Thompson et al., 2006). Generally poorer performance might be a consequence of fewer neurons tuned to slow speeds than fast speeds, as found in the primate brain (Hadad et al., 2011, Liu and Newsome, 2003). We were particularly interested, however, in how internal noise and sampling changed with age, and how these age-related effects might vary between speed conditions. Internal noise levels reduced more gradually in the slow (1.5°/s) condition than the fast (6°/s) condition, whereas sampling followed a similar rate of development for slow and fast stimuli. Furthermore, sampling appeared to follow a more protracted rate of development than that of internal noise. Internal noise reached adult-like levels by approximately 7 years of age, while sampling reached adult-like levels at a later age. Indeed, sampling was adult-like by 9 years in the fast condition, but was not yet adult-like by 11 years in the slow condition.

Our results complement a recent study by Bogfjellmo et al. (2014), which found increased sampling of direction information between the ages of 6 and 17 years for stimulus speeds of 2.8°/s and 9.8°/s, while levels of internal noise remained stable. Taken together, the current results and those of Bogfjellmo et al. suggest that internal noise reduces to adult-like levels by approximately 6–7 years, while age-related changes in sampling follow a more extended trajectory. Our finding that internal noise reduces with age echoes a previous study in the auditory domain which reported higher internal noise in children aged 6–11 years compared to adults (Buss et al., 2006), as well as reports of increased levels of internal noise in infants (Skoczenski and Norcia, 1998).

The equivalent noise method gives us an estimate of the total amount of internal noise, whilst remaining agnostic about its precise source. However, we speculate that high levels of internal noise in our direction integration task may reflect immaturity in the responses of direction-sensitive cells in V1. Specifically, imprecision in estimating the directions of local elements may be due to broad bandwidths of V1 neurons in children below the age of 7 years, which later narrow with development (at least in the primate brain, Hatta et al., 1998). Conversely, developmental increases in sampling may reflect the development of neurons in higher areas of the motion processing hierarchy thought to be involved in integrating local motion signals, such as MT/V5 (Born and Tootell, 1992, Britten et al., 1992). While MT neurons are responsive to direction information and myelinated at birth in primates (Flechsig, 1901; Movshon et al., 2004), they show immaturities in their integrative properties (Movshon et al., 2004), which could underlie the extended development of sampling reported here. Furthermore, the fact that internal noise matures before sampling corroborates neurophysiological research showing that V1 matures earlier than extrastriate areas (Distler et al., 1996; Gogtay et al., 2004; Hou et al., 2009; Kourtzi et al., 2006), which has been linked to differences in synaptic pruning (Distler et al., 1996; Gogtay et al., 2004). Future work combining psychophysical and neurophysiological measures is necessary to determine the precise neural substrate for these effects.

Our findings of age-related reductions in internal noise contrast sharply with McIntosh et al.’s (2008) report of *increasing* neural noise measured by intra-participant EEG variability between the ages of 8 and 12 years. McIntosh et al. suggested that increasing neural noise reflected the brain's increasing complexity with age, allowing one to explore multiple states and adapt to different situations. This sort of complexity, however, is not being tapped by the visual integration task used here, and instead, we refer to internal noise as uncertainty in the coding of local motion directions. Indeed, there are many different sources of noise within the nervous system (Faisal et al., 2008) and it is possible that noise may have different effects at different levels of the cortical hierarchy. However, current computational and neural models of noise are based on animal and human adult brains. It therefore remains a challenge to determine exactly how these models should be applied to the developing brain. The discrepancy between our results and those of McIntosh et al. highlight the importance of specifying what is meant by noise and the level at which it is thought to have an effect when constructing developmental models.

Our findings add to a body of literature showing a relatively protracted development of sensitivity to coherent motion (Gunn et al., 2002, Hadad et al., 2011). Our results suggest that motion coherence thresholds reach adult-like levels by approximately 9 years, which is slightly earlier than previous accounts that have suggested that maturity is reached by 10–11 years of age (Gunn et al., 2002), or 12–14 years of age (Hadad et al., 2011). Discrepancies in the age at which adult-like levels are reached are likely to be due to differences in a range of stimulus parameters (Narasimhan and Giaschi, 2012). Our study also allowed us to test the suggestion that motion coherence sensitivity may mature at different rates for different speeds. While Hadad et al. did not find a significant difference in the rates of development for sensitivity to coherent motion at 4°/s and 18°/s, Hayward et al. (2011) reported a more gradual rate of development for motion-defined form coherence thresholds at a much slower speed of 0.1°/s compared to faster speeds. While our results suggest similar rates of development for coherent motion sensitivity at a slow (1.5°/s) and faster (6°/s) speed, we noted two differences between these speed conditions: first that internal noise develops more gradually for slow speeds, and second, that sampling matures later for slow speeds than fast speeds. It is possible that such differences may have contributed to the differential rates of development in coherent motion sensitivity reported in previous studies.

The current study not only describes motion coherence thresholds but also critically helps us to understand what might limit motion coherence sensitivity during development, providing the first evidence of a commonly held assumption. We have shown here that age-related improvements in motion coherence sensitivity are driven by an increase in the effective number of local motion signals that are averaged (i.e., improved global integration). In contrast, internal noise does not limit motion coherence thresholds, at least from 5 years of age. This echoes a recent study by Falkenberg et al. (2014) showing that sampling, and not internal noise, limits the development of sensitivity to the direction of grating stimuli in children aged 5–14 years. It remains an open question, however, as to whether internal noise might limit motion sensitivity earlier on in development. It is not clear exactly how much internal noise would be needed to limit motion coherence thresholds, as there is no ideal observer model for motion coherence. Dakin et al. (2005) showed that adult observers have higher motion coherence thresholds than would be expected based on estimates of internal noise and sampling alone, which may be due to the additional requirement of segregating signal from noise dots in motion coherence tasks. The ability to extract signal from noise may be another limiting factor on motion coherence sensitivity in development, alongside sampling.

Future work should investigate whether our findings of age-related reductions in internal noise and increases in sampling generalise to different integration tasks, such as orientation integration (e.g., Dakin, 2001), particularly in light of the suggestion that dorsal and ventral processing streams may follow different developmental trajectories (e.g., Braddick et al., 2003). It is also possible that the integration reported here may relate more generally to the ability to average across other types of information, such as multisensory cues, which is also immature in childhood (e.g., Gori et al., 2008, Nardini et al., 2008, Nardini et al., 2010).

We measured fixations in a subset of participants, allowing us to establish whether age-related changes in internal noise and sampling were related to differences in the ability to maintain fixation between age groups. Interestingly, fixation stability was related to internal noise, with higher internal noise levels being associated with lower fixation stabilities. A link between eye movements and internal noise has previously been established in a study of people with albinism (Neveu et al., 2009), which reported higher levels of internal noise in participants with associated optokinetic nystagmus than those without nystagmus. Neveu et al. suggested that abnormal eye movements change the structure of visual information entering the system, which therefore disrupts the ability of the motion pathways to form normally. In our current study, the nature of the relationship between fixation stability and internal noise is unclear. It is possible that unstable eye movements limit the precision with which each individual dot's direction can be estimated by young children, or it could be that internal noise estimates and unstable fixation are both indices of increased neural variability. Also, our fixation data suggest that observers use comparable fixation strategies under conditions of low and high noise, which goes some way to support the assumption of noise-invariance of sampling in the equivalent noise model.

The current study used a paradigm that allowed us to distinguish local and global contributions to motion integration in development–a paradigm that is far more informative than standard motion coherence tasks. The equivalent noise paradigm has been successfully used to study a range of conditions in adult populations, such as amblyopia (Hess et al., 2006), glaucoma (Falkenberg and Bex, 2007), migraine (Wagner et al., 2010, Tibber et al., 2014), albinism (Neveu et al., 2009) and ageing (Arena et al., 2013, Bocheva et al., 2013, Pardhan, 2004, Pardhan et al., 1996). The quick, efficient method employed here made it feasible to estimate the internal noise and sampling of children. While it is possible that the reduced number of trials may add some measurement error, we have demonstrated that the method is still clearly sensitive to age-related changes. The equivalent noise paradigm therefore has potential applications for investigating atypical development in childhood. Motion coherence difficulties have been found in many developmental conditions, such as autism (e.g., Manning et al., 2013, Milne et al., 2002, Pellicano et al., 2005), dyslexia (Hansen et al., 2001), Fragile X syndrome (Kogan et al., 2004), and Williams Syndrome (Atkinson et al., 1997) and hence accounts of impaired global motion processing in these conditions lack specificity. Indeed, it could be that reduced coherent motion sensitivity is a general consequence of atypical brain development (Braddick et al., 2003), or it could be that atypical motion coherence thresholds arise from different causal factors in different conditions (see Pellicano and Gibson, 2008). The equivalent noise paradigm therefore offers the potential to identify what might contribute to elevated motion coherence thresholds across different conditions.

## 5 Conclusions

In sum, this study enriches our understanding of the development of visual motion processing by characterising local and global limits to direction integration. Internal noise levels reduce through childhood, while the number of samples integrated increases. Furthermore, the gradual development of motion coherence sensitivity with age appears to be due to improvements in sampling rather than reductions in internal noise. The current study provides a useful platform for investigating atypical development of global motion perception abilities in conditions such as autism, as well as informing future models of development incorporating ‘noise’.

## Conflict of interest

No authors have any conflicts of interest to declare.

## References

[bib0005] Adams R.J., Courage M.L. (2002). Using a single test to measure human contrast sensitivity from early childhood to maturity. Vision Res..

[bib0010] Ahmed I.J., Lewis T.L., Ellemberg D., Maurer D. (2005). Discrimination of speed in 5-year-olds and adults: are children up to speed?. Vision Res..

[bib0015] Arena A., Hutchinson C.V., Shimozaki S.S., Long M.D. (2013). Visual discrimination in noise: behavioural correlates of age-related cortical decline. Behav. Brain Res..

[bib0020] Atkinson J., King J., Braddick O., Nokes L., Anker S., Braddick F. (1997). A specific deficit of dorsal stream function in Williams’ syndrome. Neuroreport.

[bib0025] Barlow H., Tripathy S.P. (1997). Correspondence noise and signal pooling in the detection of coherent visual motion. J. Neurosci..

[bib0030] Barlow H.B. (1956). Retinal noise and absolute threshold. J. Opt. Soc. Am..

[bib0035] Beck J.M., Ma W.J., Pitkow X., Latham P.E., Pouget A. (2012). Not noisy, just wrong: the role of suboptimal inference in behavioral variability. Neuron.

[bib0040] Bocheva N., Angelova D., Stefanova M. (2013). Age-related changes in fine motion direction discriminations. Exp. Brain Res..

[bib0845] Bogfjellmo L.-G., Bex P.J., Falkenberg H.K. (2014). The development of global motion discrimination in school aged children. J. Vis..

[bib0045] Born R.T., Tootell R.B.H. (1992). Segregation of global and local motion processing in primate middle temporal visual area. Nature.

[bib0050] Braddick O.J., Atkinson J., Wattam-Bell J. (2003). Normal and anomalous development of visual motion processing: motion coherence and ‘dorsal-stream vulnerability’. Neuropsychologia.

[bib0055] Brainard D.H. (1997). The psychophysics toolbox. Spat. Vis..

[bib0060] Britten K.H., Shadlen M.N., Newsome W.T., Movshon J.A. (1992). The analysis of visual motion: a comparison of neuronal and psychophysical performance. J. Neurosci..

[bib0065] Burr D., Thompson P. (2011). Motion psychophysics: 1985–2010. Vision Res..

[bib0070] Burr D.C., Fiorentini A., Morrone C. (1998). Reaction time to motion onset of luminance and chromatic gratings is determined by perceived speed. Vision Res..

[bib0075] Buss E., Hall J.W., Grose J.H. (2006). Development and the role of internal noise in detection and discrimination thresholds with narrow band stimuli. J. Acoust. Soc. Am..

[bib0080] Dakin S., Frith U. (2005). Vagaries of visual perception in autism. Neuron.

[bib0085] Dakin S.C. (2001). Information limit on the spatial integration of local orientation signals. J. Opt. Soc. Am. A.

[bib0090] Dakin S.C., Mareschal I., Bex P.J. (2005). Local and global limitations on direction integration assessed using equivalent noise analysis. Vision Res..

[bib0095] Delaney H.D., Maxwell S.E. (1981). On using analysis of covariance in repeated measures designs. Multivar. Behav. Res..

[bib0800] Distler C., Bachevalier J., Kennedy C., Mishkin M., Ungerleider L.G. (1996). Functional development of the corticocortical pathway for motion analysis in the macaque monkey: a 14C-2-deoxyglucose study. Cereb. Cortex.

[bib0100] Edwards M., Badcock D.R., Smith A.T. (1998). Independent speed-tuned global-motion systems. Vision Res..

[bib0105] Ellemberg D., Lewis T.L., Liu C.H., Maurer D. (1999). Development of spatial and temporal vision during childhood. Vision Res..

[bib0110] Faisal A.A., Selen L.P.J., Wolpert D.M. (2008). Noise in the nervous system. Nat. Rev. Neurosci..

[bib0115] Falkenberg H.K., Bex P.J. (2007). Sources of motion-sensitivity loss in glaucoma. Invest. Ophthalmol. Vis. Sci..

[bib0120] Falkenberg H.K., Simpson W.A., Dutton G.N. (2014). Development of sampling efficiency and internal noise in motion detection and discrimination in school-aged children. Vision Res.

[bib0825] Flechsig P. (1901). Developmental (myelogenetic) localisation of the cerebral cortex in the human subject. The Lancet.

[bib0125] Fox M.D., Snyder A.Z., Vincent J.L., Raichle M.E. (2007). Intrinsic fluctuations within cortical systems account for intertrial variability in human behavior. Neuron.

[bib0530] Gogtay N., Giedd J.N., Lusk L., Hayashi K.M., Greenstein D., Vaituzis A.C. (2004). Dynamic mapping of human cortical development during childhood through early adulthood. Proc. Natl. Acad. Sci. U.S.A..

[bib0630] Garey L.J., de Courten C. (1983). Structural development of the lateral geniculate nucleus and visual cortex in monkey and man. Behav. Brain Res..

[bib0130] Gori M., Del Viva M., Sandini G., Burr D.C. (2008). Young children do not integrate visual and haptic form information. Curr. Biol..

[bib0135] Gunn A., Cory E., Atkinson J., Braddick O., Wattam-Bell J., Guzzetta A., Cioni G. (2002). Dorsal and ventral stream sensitivity in normal development and hemiplegia. Neuroreport.

[bib0140] Hadad B.-S., Maurer D., Lewis T.L. (2011). Long trajectory for the development of sensitivity to global and biological motion. Dev. Sci..

[bib0145] Hansen P.C., Stein J.F., Orde S.R., Winter J.L., Talcott J.B. (2001). Are dyslexics’ visual deficits limited to measures of dorsal stream function?. Neuroreport.

[bib0150] Hatta S., Kumagami T., Qian J., Thornton M., Smith E.L., Chino Y.M. (1998). Nasotemporal directional bias of V1 neurons in young infant monkeys. Invest. Ophthalmol. Vis. Sci..

[bib0155] Hayward J., Truong G., Partanen M., Giaschi D. (2011). Effects of speed, age, and amblyopia on the perception of motion-defined form. Vision Res..

[bib0160] Hess R.F., Mansouri B., Dakin S.C., Allen H.A. (2006). Integration of local motion is normal in amblyopia. J. Opt. Soc. Am. A.

[bib0565] Hou C., Gilmore R.O., Pettet M.W., Norcia A.M. (2009). Spatio-temporal tuning of coherent motion evoked responses in 4–6 month old infants and adults. Vision Res..

[bib0665] Hubel D.H., Wiesel T.N. (1962). Receptive fields, binocular interaction and functional architecture in the cat's visual cortex, *J*. Physiol..

[bib0765] Huttenlocher P.R., de Courten C. (1987). The development of synapses in striate cortex of man. Human Neurobiol..

[bib0865] Huttenlocher P.R., de Courten C., Garey L.J., Van der Loos H. (1982). Synaptogenesis in human visual cortex—evidence for synapse elimination during normal development. Neurosci. Lett..

[bib0165] Kleiner M., Brainard D.H., Pelli D.G. (2007). What's new in Psychtoolbox-3?. Perception.

[bib0170] Kogan C.S., Bertone A., Cornish K., Boutet I., Der Kaloustian V.M., Andermann E., Chaudhuri A. (2004). Integrative cortical dysfunction and pervasive motion perception deficit in fragile X syndrome. Neurology.

[bib0175] Kourtzi Z., Augath M., Logothetis N.K., Movshon J.A., Kiorpes L. (2006). Development of visually evoked cortical activity in infant macaque monkeys studied longitudinally with fMRI. Magn. Reson. Imaging.

[bib0180] Liu J., Newsome W.T. (2003). Functional organization of speed tuned neurons in visual area MT. J. Neurophysiol..

[bib0185] Manning C., Aagten-Murphy D., Pellicano E. (2012). The development of speed discrimination abilities. Vision Res..

[bib0190] Manning C., Charman T., Pellicano E. (2013). Processing slow and fast motion in children with autism spectrum conditions. Autism Res..

[bib0195] McIntosh A.R., Kovacevic N., Itier R.J. (2008). Increased brain signal variability accompanies lower behavioral variability in development. PLoS Comput. Biol..

[bib0870] Mikami A., Newsome W.T., Wurtz R.H. (1986). Motion selectivity in macaque visual cortex. II. Spatiotemporal range of directional interactions in MT and V1. J. Neurophysiol..

[bib0200] Milne E., Swettenham J., Hansen P., Campbell R., Jeffries H., Plaisted K. (2002). High motion coherence thresholds in children with autism. J. Child Psychol. Psychiatry.

[bib0805] Milner A.D., Goodale M.A. (1995).

[bib0905] Movshon J.A., Rust N.C., Kohn A., Kiorpes L., Hawken M.J. (2004). Receptive-field properties of MT neurons in infant macaques. Perception.

[bib0205] Narasimhan S., Giaschi D. (2012). The effect of dot speed and density on the development of global motion perception. Vision Res..

[bib0210] Nardini M., Bedford R., Mareschal D. (2010). Fusion of visual cues is not mandatory in children. Proc. Natl. Acad. Sci. U. S. A..

[bib0215] Nardini M., Jones P., Bedford R., Braddick O. (2008). Development of cue integration in human navigation. Curr. Biol..

[bib0220] Neveu M.M., Jeffery G., Moore A.T., Dakin S.C. (2009). Deficits in local and global motion perception arising from abnormal eye movements. J. Vis..

[bib0225] Newsome W.T., Paré E.B. (1988). A selective impairment of motion perception following lesions of the middle temporal visual area (MT). J. Neurosci..

[bib0230] Pardhan S. (2004). Contrast sensitivity loss with aging: sampling efficiency and equivalent noise at different spatial frequencies. J. Opt. Soc. Am. A.

[bib0235] Pardhan S., Gilchrist J., Elliott D.B., Beh G.K. (1996). A comparison of sampling efficiency and internal noise level in young and old subjects. Vision Res..

[bib0240] Parrish E.E., Giaschi D.E., Boden C., Dougherty R. (2005). The maturation of form and motion perception in school age children. Vision Res..

[bib0245] Pelli D.G., Blakemore C. (1990). Vision: Coding and Efficiency.

[bib0250] Pelli D.G. (1997). The VideoToolbox software for visual psychophysics: transforming numbers into movies. Spat. Vis..

[bib0255] Pellicano E., Gibson L., Maybery M., Durkin K., Badcock D.R. (2005). Abnormal global processing along the dorsal visual pathway in autism: a possible mechanism for weak visuospatial coherence?. Neuropsychologia.

[bib0260] Pellicano E., Gibson L.Y. (2008). Investigating the functional integrity of the dorsal visual pathway in autism and dyslexia. Neuropsychologia.

[bib0265] Skoczenski A.M., Norcia A.M. (1998). Neural noise limitations on infant visual sensitivity. Nature.

[bib0270] Thomas M.S.C., Annaz D., Ansari D., Scerif G., Jarrold C., Karmiloff-Smith A. (2009). Using developmental trajectories to understand developmental disorders. J. Speech Lang. Hear. Res..

[bib0275] Thompson P., Brooks K., Hammett S.T. (2006). Speed can go up as well as down at low contrast: implications for models of motion perception. Vision Res..

[bib0280] Tibber M.S., Kelly M.G., Jansari A., Dakin S.C., Shepherd A.J. (2014). An inability to exclude visual noise in migraine. Invest. Ophthalmol. Vis. Sci..

[bib0285] Wagner D., Manahilov V., Loffler G., Gordon G.E., Dutton G.N. (2010). Visual noise selectively degrades vision in migraine. Invest. Ophthalmol. Vis. Sci..

[bib0290] Watson A.B., Pelli D.G. (1983). QUEST: a Bayesian adaptive psychometric method. Percept. Psychophys..

[bib0295] Wattam-Bell J. (1991). The development of motion-specific cortical responses in infants. Vision Res..

[bib0300] Wattam-Bell J. (1992). The development of maximum displacement limits for discrimination of motion direction in infancy. Vision Res..

[bib0885] Webster K.E., Dickinson J.E., Battista J., McKendrick A.M., Badcock D.R. (2011). Increased internal noise cannot account for motion coherence processing deficits in migraine. Cephalalgia.

[bib0305] Williams B.R., Hultsch D.F., Strauss E.H., Hunter M.A., Tannock R. (2005). Inconsistency in reaction time across the life span. Neuropsychology.

